# “METAGENOTE: a simplified web platform for metadata annotation of genomic samples and streamlined submission to NCBI’s sequence read archive”

**DOI:** 10.1186/s12859-020-03694-0

**Published:** 2020-09-03

**Authors:** Mariam Quiñones, David T. Liou, Conrad Shyu, Wongyu Kim, Ivan Vujkovic-Cvijin, Yasmine Belkaid, Darrell E. Hurt

**Affiliations:** 1 Bioinformatics and Computational Biosciences Branch, Office of Cyber Infrastructure and Computational Biology, National Institute of Allergy and Infectious Diseases, National Institutes of Health, Bethesda, MD 20892 USA; 2 Metaorganism Immunity Section, Laboratory of Immune System Biology, National Institute of Allergy and Infectious Diseases, National Institute of Health, Bethesda, MD 20292 USA

**Keywords:** Metadata, Sequence read archive, Ontologies, Genomic samples, Web platform

## Abstract

**Background:**

The improvements in genomics methods coupled with readily accessible high-throughput sequencing have contributed to our understanding of microbial species, metagenomes, infectious diseases and more. To maximize the impact of these genomics studies, it is important that data from biological samples will become publicly available with standardized metadata. The availability of data at public archives provides the hope that greater insights could be obtained through integration with multi-omics data, reproducibility of published studies, or meta-analyses of large diverse datasets. These datasets should include a description of the host, organism, environmental source of the specimen, spatial-temporal information and other relevant metadata, but unfortunately these attributes are often missing and when present, they show inconsistencies in the use of metadata standards and ontologies.

**Results:**

METAGENOTE (https://metagenote.niaid.nih.gov) is a web portal that greatly facilitates the annotation of samples from genomic studies and streamlines the submission process of sequencing files and metadata to the Sequence Read Archive (SRA) (Leinonen R, et al, Nucleic Acids Res, 39:D19-21, 2011) for public access. This platform offers a wide selection of packages for different types of biological and experimental studies with a special emphasis on the standardization of metadata reporting. These packages follow the guidelines from the MIxS standards developed by the Genomics Standard Consortium (GSC) and adopted by the three partners of the International Nucleotides Sequencing Database Collaboration (INSDC) (Cochrane G, et al, Nucleic Acids Res, 44:D48-50, 2016) - National Center for Biotechnology Information (NCBI), European Bioinformatics Institute (EBI) and the DNA Data Bank of Japan (DDBJ). METAGENOTE then compiles, validates and manages the submission through an easy-to-use web interface minimizing submission errors and eliminating the need for submitting sequencing files via a separate file transfer mechanism.

**Conclusions:**

METAGENOTE is a public resource that focuses on simplifying the annotation and submission process of data with its corresponding metadata. Users of METAGENOTE will benefit from the easy to use annotation interface but most importantly will be encouraged to publish metadata following standards and ontologies that make the public data available for reuse.

## Background

Genomic research is facilitating our understanding of genetic and epigenetic variation, species diversity, transcription and gene regulation, metagenomics and other applications, all of which rely heavily on the generation of large sequencing datasets. Policies such as the NIH Data Sharing policy have been established with the goal of ensuring that researchers share the raw sequencing files produced in these studies to facilitate replication of findings, to enable discoveries beyond those reported in the original study, and to allow for meta-analyses that aggregate data from multiple studies. In fact, the number of datasets published at the SRA [1] has been on a steady increase and doubling every 18–24 months [2]. The expectation of the scientific community is that these publicly available datasets could be leveraged for integration with additional studies and for meta-analyses that will find deeper biological insights or even be used for detecting experimental sources of bias in the data. A meta-analysis requires the availability of sufficient metadata to describe the sample and associated files but unfortunately most datasets end up getting published with the minimum required metadata and/or inconsistent use of vocabulary.

In the case of metagenomics studies, the metadata annotation is critical for describing the sample source, tissue collection method, environment and additional details such as DNA extraction method or sequence library preparation, all of which could potentially impact microbial survey results. To address the need for standardized metadata collection, the genomic community has adopted the use of the Genomic Standards Consortium [3] minimal information standards (MIxS), in particular the MIMARKS (marker genes), MIGS (genome sequence), MIMS (metagenome sequence) and more recently the checklists MIMAG (metagenome assembled genomes), MISAG (single amplified genome) and MIUVIG (uncultivated virus genome) available in the format of downloadable spreadsheets [4]. These standards require the use of structured vocabulary derived from specific ontologies including the Environmental Ontology (ENVO), Chemical Entities of Biological Interest (ChEBI), Foundations of Medical Anatomy (FMA) and Experimental Factor Ontology (EFO) for specific attributes [5–8]. Currently, many researchers in the microbiome field publish raw metadata by uploading metadata files directly to the SRA using the SRA Submission Portal or to the European Nucleotide Archive (ENA) using the ENA Webin tool, the latter of which also provides an optional metagenomics analysis pipeline service through MGnify. Alternatively, researchers can upload files to MG-RAST or QIITA, which in addition to facilitating release of data to INSDC databases [9] (through ENA), provide metagenomics processing and analysis pipelines [10–13]. The submission portals for these archives and the tools mentioned provide the option to use MIxS-based packages but not all require users to strictly adhere to the requirements of the MIxS checklists. While the lack of enforcement of standards makes it easier for the submitter to publish the data, these could be missing key metadata attributes, thereby reducing their usability. This has been termed as “metadata gap” by the project GeOMe [14], which provides a web portal for storing and querying geographic and ecological metadata in a format compatible with MixS and TDWG’s Darwin Core standards [15].

In addition to enforcing the use of standards, the inconsistent use of vocabulary needs to be addressed in order to facilitate queries and cross-study analyses. Ideally, researchers would easily find appropriate ontology terms to use when annotating studies. To streamline this process, there have been several Google Docs Add-ons such as OntoMaton and Webulous, which were designed to facilitate searching and importing ontology terms directly from within the spreadsheets [16, 17]. Another step forward can be seen in the CEDAR workbench [18], which was designed to allow users to create custom templates with numerous integrated ontologies and can be configured to export a file in the format required for submission of metadata to archives. While CEDAR has great potential, it does require an initial configuration effort, which is not trivial.

In spite of the efforts by the multiple initiatives listed above, many researchers still find it cumbersome to create and use the standard templates for annotation, fill in the required minimum metadata annotation established by archives and finally submit metadata along with associated raw sequencing files to repositories using the recommended transfer protocols. As a consequence, even genomics studies with public data files tend to have very limited metadata available. For example, in the NCBI BioSample database, as of the moment of writing this article, only 3.6% of samples submitted using the MIGS.ba.host-associated.5.0 package included metadata to describe the optional attribute “host tissue sampled” and only 18% of samples published using the MIMARKS.specimen.host associated package included metadata for the optional attribute “host diet”. In addition, a count of the most commonly used metadata terms for the required attribute “developmental stage” in the package model.organism.animal, shows multiple redundant terms (e.g. “Adult”, “mature”) and extensive use of words “not applicable”, “not collected” and “missing” (see Table 1). To address the needs for richer and unified metadata, this manuscript describes METAGENOTE, a web tool that streamlines metadata annotation, enforces the use of MIxS standards and recommended ontologies and finally automates submission to NCBI BioProject, BioSample and SRA, all from a user-friendly web interface.

**Table 1:** Example of metadata reported for selected attributes in BioSample database. The attribute chosen for counting is shown in bold. The words entered by users are shown next to the number of times it was found in the subset of the samples examined

**Package:**	**MIGS.ba.host-associated.5.0**		**MIMARKS.specimen.host-associated.5.0**		**Model.organism.animal.1.0**	
Total entries:	2598		19,338		276,262	
**Attribute:**	**host tisssue sampled**		**host diet**		**developmental stage (required)**	
Number of responses	94		3616		179,373	
Metadata provided	27	intestine	358	herbivore	41,112	adult
	16	gill	206	CHO	18,309	missing
	8	Gill	199	therapeutic_hydrolyzed_protein_diet	16,856	not collected
	6	spleen	191	Chow	16,416	Adult
	5	nasopharynx	144	Artemia_salina	16,209	not applicable
	5	Mammary gland	106	KD	5465	Juvenile
	4	Sputum	105	regulat_diet	4797	juvenile
	3	bronchoalveolar lavage	103	Unmedicated_swine_feed	2315	NA
	2	Tissue	91	LFD	2123	Embryo
	2	pharynx	90	purified_rodent_diet_AIN-93 M	2098	mixed
	2	head kidney	90	10%_sucrose_solution	1511	mixed stage
	1	Whole	88	HFD	1401	mature
	1	submandibular lymph node	88	finely_ground_Koi_food_(Foster_&amp;_Smith,_Inc._Rhinelander,_WI)	1160	larva
	1	stomach	84	10%_w/w_rice_bran_fermented_with_Bifidobacterium_longum_ATCC-55813	1077	MISSING
	1	skin lesion	79	10%_w/w_heat-stabilized_rice_bran	1014	tadpole
	1	Pleural fluid	67	Termitomyces	988	adult pig
	1	periodontal pocket	64	HC	947	months postnatal
	1	nasal cavity	64	dPD	849	embryo
	1	hepatopancreas	62	BASE	682	morula
	1	gut	60	omnivore	679	Not Collected
	1	gills	56	Fe_50ppm	607	unknown
	1	fecal droplets	55	Fe_500ppm	563	calf
	1	fat body tissues	52	dZD	546	not determined
	1	eye	50	dN	534	Not collected

### Implementation

Our main goal was to create a tool to facilitate detailed annotation of samples, encourage unification of vocabulary, and streamline submission of metadata with associated files to SRA. METAGENOTE was originally developed to meet the needs of the microbiome community but was later expanded to accommodate for annotations of any other types of genomic samples with associated sequencing data that is intended to be released publicly.

**Fig. 1:**
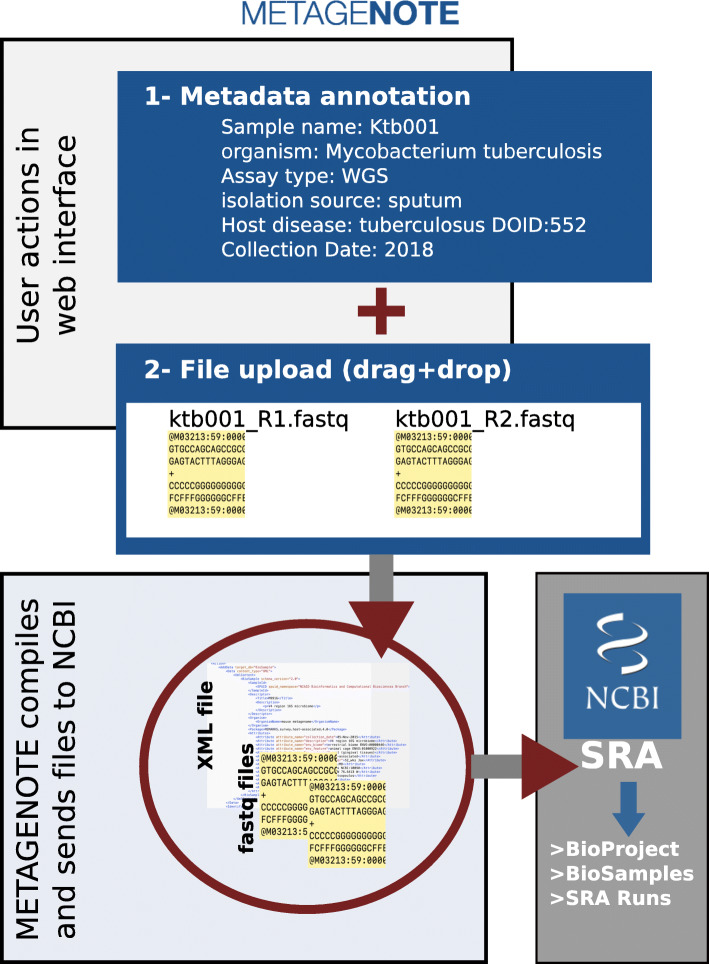
METAGENOTE Application Diagram. METAGENOTE allows annotation and transfer of files to SRA. METAGENOTE prepares an XML file in the format required by the SRA’s API to automatically create all records for BioProject, BioSamples and Runs. Sequence files are uploaded to METAGENOTE via a drag and drop action into the browser. METAGENOTE finally sends raw files and the XML file to NCBI’s server

METAGENOTE is written in Java, HTML, JavaScript and Python. It has been implemented with a RESTful web service and a MariaDB database for metadata storing. It incorporates an import functionality for a metadata table in text or Microsoft Excel format and an interface for matching user attributes with those in the standards. The user interface shows a familiar table view that allows direct entry of words, and column or row manipulations. METAGENOTE also includes a sequencing file upload mechanism, which stores files transiently and securely during the submission process and sends them via an Aspera client to the NCBI SRA server (Fig. 1).

### Unification of vocabulary use

In order to improve consistency in the use of standardized vocabulary, METAGENOTE includes suggestions of frequently used words in a drop-down menu and also an ontology word search functionality option, which provides quick retrieval of words from trimmed versions of the ontologies ENVO, ChEBI, EFO and FMA recommended by the MIxS checklists. For a few critical attributes such as the ‘host’ and ‘organism’, METAGENOTE provides suggestions taken from the NCBI Taxonomy database or from additional ontologies. The words available via the drop-down menu were originally found in the MIxS attribute description or identified after a frequency ranking of words used in public BioSample entries submitted by groups around the world. As more metadata becomes available, METAGENOTE will continue analyzing public metadata, identifying words of general applicability and whenever possible presenting the ontology equivalent to the user, thereby encouraging frequent use of ontology terms.

It is assumed that in order to maximize the possibility of data reuse, the ideal scenario would include strict validation measures for the type of package and words used. In spite of this, the METAGENOTE team decided to maintain the flexibility offered by the MIxS checklists, which recommend the use of ontologies for some attributes but allow the use of free text for other attributes. Our vision is to build a user-friendly tool that will help researchers get familiarized with the use of ontologies, will streamline the annotation and submission process and ultimately results in higher quality and quantity of metadata submitted to public repositories. Having said that, METAGENOTE, as well as other research organizations and repositories should strive to find ways of enforcing stricter measures for higher quality of metadata.

### Validation of format and completion

With the need for vocabulary validation, METAGENOTE includes functionality to notify the user of missing required metadata or errors in the format of specific attributes. It follows the requirements set by the BioSample database and validates the entries in the XML format file produced using the NCBI BioSample and SRA schema during the submission process to prevent common errors when submitting metadata. The server notifies the user via email when the submission is in progress or if there were any errors. Once the files are sent to NCBI, the user then receives emails directly from the BioSample or SRA databases with additional notifications of submission progress.

### Guest and private access

The main functionality of METAGENOTE for sample group annotation, use of ontologies, validation and submission to SRA is publicly available. Files are temporarily stored to allow for the submission process to complete. Users receive notifications from METAGENOTE and from NCBI to the email provided by the user. An additional functionality is available to private groups within our local institution (NIH), which is to store sample group annotations in a “Workspace” area. These draft tables will remain private during preparation.

## Results and discussion

### Annotation user Interface

To explore the annotation capabilities of METAGENOTE, users can view previously annotated sample groups available via the “Browse” menu option. In the short time since METAGENOTE was released publicly, it has already been used to annotate and submit data of genomic samples from *Mycobacterium tuberculosis*, *Neospora caninum*, mouse stool microbiome, human stool microbiome and others. For example, it was used to annotate and send for SRA publication a set of 211 samples from a study investigating the effect of antihelminthic treatment on the human gut bacterial microbiota (METAGENOTE ID:8A9ZD8CWZPP, BioProject ID: PRJNA510835). It was also used in the annotation of 157 samples from a study on the gut microbiota of anti-retroviral treated HIV-infected patients (see METAGENOTE ID:RKLLJ2DL8RQ or BioProject ID: PRJNA589036).

**Fig. 2:**
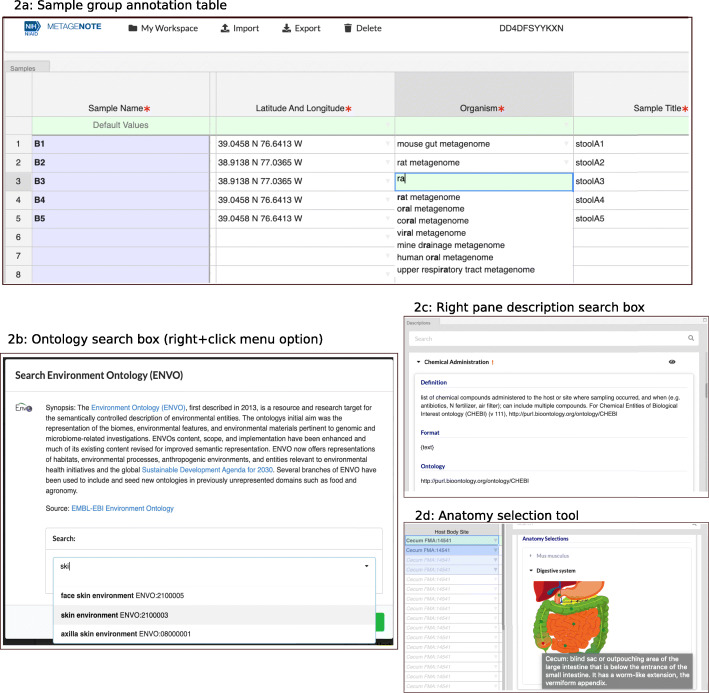
METAGENOTE simplifies sample annotation. Metadata annotation can be done by typing directly into the sample group annotation Table (2a), selecting frequently-used words available in the drop-down menu or importing words using the ontology search functionality within the right+click menu option (2b). In addition, the right pane provides a description search box (2c) to aid user in finding information of the required field or to open an anatomy selection tool (2d)

METAGENOTE provides the user with a collection of web-based packages for genomic samples derived from model organisms, microbes, environmental biomes, eukaryotes, and human cell lines. More specifically, these packages include all of the GSC’s MIxS checklists incorporated in the NCBI’s BioSample Submission Portal (MIMARKS 5.0, MIGS 5.0, MIMS 5.0, MISAG 5.0, MIMAG 5.0, MIUVID 5.0) as well as the NCBI’s Model.organism.animal 1.0 and the Human 1.0 packages [19]. For additional details on the packages available through METAGENOTE, view Table 2 and the METAGENOTE GitHub repository. Annotation is done directly on the web sample group annotation table (Fig. 2) or a user can choose to batch import metadata using an existing spreadsheet and then proceed to match custom column headers to corresponding package attributes, a process which also automatically appends the unmatched custom ones. The import feature allows users to bring their own “package” in the form of a spreadsheet file with custom attributes that when completed will be ultimately submitted to SRA along with the minimum required attributes for the related package selected. For example, a user working on SARS-CoV-2 samples that wishes to share a unique set of attributes, could first select the “Viral genome” or “Uncultivated viral genome” data source, then select the Human associated package (based on the MIxS miuvig.human-associated version 5.0) and finally proceed to import the custom spreadsheet to create a new sample group table with column headers that contain the unique attributes as well as the standard package attributes.

**Table 2:** List of packages available in METAGENOTE

	Checklists (version 5.0)	Human packages	Environmental packages	Other Packages
**GSC MIxS Standards**	MIGS for genomes	Human Associated, Human Gut, Human Oral, Human Skin, Human Vaginal	Air, Sediment, Soil, Wastewater, Water	Non-Human Animal Host, Miscellaneous, Plant Associated, Microbial
	MIMS for metagenomes			
	MIMARKS for marker genes.			
	MISAG for Single Amplified Genome			
	MIMAG for Metagenome-Assembled Genome			
	MIUVIG for Uncultivated Virus Genome			
**NCBI**	Human 1.0			
	Model Organism Animal 1.0

The right pane of the sample group table provides the attribute’s format description, lists any recommended ontology and provides examples of commonly used words for the attributes included. In order to further simplify the search for ontology terms, METAGENOTE offers a “right click” menu option to launch the ontology search functionality, which provides a keyword search box for retrieving ontology terms, this in addition to the drop-down menu options for frequently used words and the Anatomy Selection Diagram available for specific host tissue or body site attributes (Fig. 2). As described above, the current version of METAGENOTE harbors trimmed versions of four main ontologies but users can also find the URLs to web servers for searching the full ontologies in the right Descriptions pane. Future enhancements to the application should focus on integration of additional ontologies and improvements on the validation of the proper use of the standards.

### Connectivity with SRA

A great advantage of METAGENOTE is that it links directly to SRA via an API, which receives an XML file containing all the sample metadata and names of associated files and automatically triggers the creation of NCBI BioProject, BioSample and SRA Experiment records. METAGENOTE guest and registered users follow a guided submission workflow, which involves “drag and drop” of files into the browser box and pairing of sample names with uploaded corresponding raw sequencing files. These files get transferred from their temporary secured server location to the NCBI server via Aspera for high speed file transfer as instructed by SRA. In the last step of the submission, the user enters general study metadata such as the Title and Description, which then appears in the BioProject page. METAGENOTE has various levels of validation to prevent errors during submission and it also provides the user with email notifications when submission is in progress. Once the submission has been accepted and published by SRA, the data will also become accessible from the EBI and DDBJ sites following the data-exchange convention previously established by the INSDC.

## Conclusions

METAGENOTE’s focus is on improving the quality of metadata available to the public by simplifying the metadata annotation and submission process. In the process, it will educate researchers on the importance of using standards and structured vocabulary by bringing these standards to them in simple web table format with suggestions of frequently used terms or ontologies. METAGENOTE users are finding the user-interface very simple for submission of files to SRA, which will provide richer and more standardized metadata to the public archives.

While METAGENOTE allows for creation of tables starting from an empty sample group annotation table, it also serves as a complement to analysis tools that require an initial metadata table. For example, for microbiome analysis, researchers could first run analysis pipelines using the Nephele platform [20] and then use the same Nephele mapping file as the input file for METAGENOTE, thereby importing sample IDs and other metadata for all samples used in the analysis.

The scientific community is now gaining awareness of the need to make data publicly available in accordance with the FAIR principles [21]. The problem is that public metadata might be accessible but not always findable and reusable because they lack completeness, accuracy, and consistency in the metadata [22]. METAGENOTE not only ensures that the MIxS packages are used to provide completeness but facilitates accuracy and consistency through giving access to ontologies and suggestions on frequently used words.

## Data Availability

http://github.com/niaid/metagenote

## Abbreviations

SRA: Sequence Read Archive
MIxS: Minimum information for any (x) sequence
GSC: Genomics Standard Consortium
INSDC: International Nucleotides Sequencing Database Collaboration
NCBI: National Center for Biotechnology Information
EBI: European Bioinformatics Institute
DDBJ: DNA Data Bank of Japan
NIH: National Institutes of Health
MIMARKS: Minimum Information about a Marker Gene Sequence
MIMS: Minimum Information about a Metagenome Sequence
MIGS: Minimum Information about a Genome Sequence
MISAG: Minimum Information about a Single Amplified Genome
MIUVIG: Minimum Information about an Uncultivated Virus Genome
ENVO: Environmental Ontology
ChEBI: Chemical Entities of Biological Interest
FMA: Foundations of Medical Anatomy
EFO: Experimental Factor Ontology
ENA: European Nucleotide Archive
MG-RAST: Metagenomics RAST Server
GeOMe: Genomics Observatories Meta-Database
TDWG: Taxonomic Databases Working Group; today’s Biodiversity Information Standards
CEDAR: Center for Expanded Data Annotation and Retrieval
XML: Extensible Markup Language
SARS-CoV-2: severe acute respiratory syndrome coronavirus 2
API: Application Programming Interface
